# Transformation of terahertz vibrational modes of cytosine under hydration

**DOI:** 10.1038/s41598-020-67179-z

**Published:** 2020-06-24

**Authors:** Donggun Lee, Hwayeong Cheon, Seo-Yeon Jeong, Joo-Hiuk Son

**Affiliations:** 1 Department of Physics, University of Seoul, Seoul, 02504 Republic of Korea; 2 Biomedical Engineering Research Center, Asan Medical Center, 88, Olympic-ro 43-gil, Songpa-gu, Seoul, Seoul, 05505 Republic of Korea

**Keywords:** Optical spectroscopy, Computational chemistry

## Abstract

Cytosine and cytosine monohydrate are representative biomolecules for investigating the effect of hydrogen bonds in deoxyribonucleic acid. To better understand intermolecular interactions, such as hydrogen bonds, between nucleobases it is necessary to identify the low-frequency vibrational modes associated with intermolecular interactions and crystalline structures. In this study, we investigated the characteristic low-frequency vibrational modes of cytosine and cytosine monohydrate using terahertz time-domain spectroscopy (THz-TDS). The crystal geometry was obtained by the powder X-ray diffraction technique. The optimized atomic positions and the normal modes in the terahertz region were calculated using density functional theory (DFT), which agreed well with the experimental results. We found that overall terahertz absorption peaks of cytosine and cytosine monohydrate consist of collective vibrations mixed with intermolecular and intramolecular vibrations in mode character analysis, and that the most intense peaks of both samples involve remarkable intermolecular translational vibration. These results indicate that THz-TDS combined with DFT calculations including mode character analysis can be an effective method for understanding how water molecules contribute to the characteristics of the low-frequency vibrational modes by intermolecular vibrations with hydrogen bonding in biological and biomedical applications.

## Introduction

Cytosine (C) is the fundamental genetic unit of the nucleobases of deoxyribonucleic acid (DNA). It is composed of a pyrimidine ring, with an amine group and a ketone group that interacts with guanine in DNA through a hydrogen bonds. It is important to understand the properties of nucleobases because these molecules are the elemental units that influence biological characteristics such as genes, transcription, translation, and replication. Since C is also used to synthesize various nucleoside and nucleotide molecules, as the main pyrimidines in DNA^1^, it is important to examine the unexpected ingredients in the C structure. Anhydrous C can transform into a hydrated crystal with water molecules and is called cytosine monohydrate (C-MH), the crystal structure of which was first studied in 1963^2^. C-MH was studied to investigate the C tautomers that lead to the disability of the biological functions of the DNA base^3^, the effect of ionizing radiation on DNA^4^, and to understand the oxidative damage in DNA^5^ because water molecules greatly influence DNA bases as a hydrogen-bonding donor and acceptor. As the main model for DNA bases, investigating the different low-frequency spectra of C and C-MH is necessary not only to identify unexpected hydration in the pharmaceutical industry but also to clearly identify how water molecules influence the DNA base under hydrogen bonding.

The dynamics of water molecules have significant importance in biology and chemistry. As a result, they have been studied in the terahertz (THz) frequency region, such as the Debye relaxation process of liquid water^6,7^, the retardation of water dynamics with a solute^8^, and the dynamics of hydration water around protein^9^. The THz frequency range is associated with the low-frequency vibrational modes, including the collective vibrations of intermolecular and intramolecular interactions^10^, the phonon modes of crystalline molecules^11^, and in-plane and out-of-plane bending vibrations of many molecules^12^. THz time-domain spectroscopy (THz-TDS) has been utilized in the study of such vibrational characteristics of biomolecules^13–18^, as well as in identifying biological functions^19–21^, and in applications to biomedical, pharmaceutical, and clinical industries^22–30^. Moreover, THz-TDS has been employed to investigate the low-frequency vibrational modes of C and C-MH, because this technique is sensitive to intermolecular interactions, such as hydrogen bonds dependent on molecular conformations and environments.

In this study, we present the THz spectra of C and C-MH to indicate the characteristic influence of water on C and demonstrate that intermolecular vibrations significantly contribute to the THz absorption peaks. The distinct characteristic peaks of C and C-MH were obtained in the absorption spectra at room temperature, using the THz-TDS system. The crystalline nature inducing the different THz spectra of the two molecules was distinguished by powder X-ray diffraction (PXRD) pattern analysis. Additionally, the experimentally acquired THz spectra were compared to the calculation results of the normal modes, acquired using density functional theory (DFT). Each normal mode was decomposed into intermolecular and intramolecular vibrations using mode characteristic analysis. Based on our experimental and theoretical results, we show the low-frequency vibrational mode characteristics of C and C-MH, as well as how water molecules contribute to intermolecular vibrations.

## Results

### THz absorption spectra of C and C-MH

**Figure 1:**
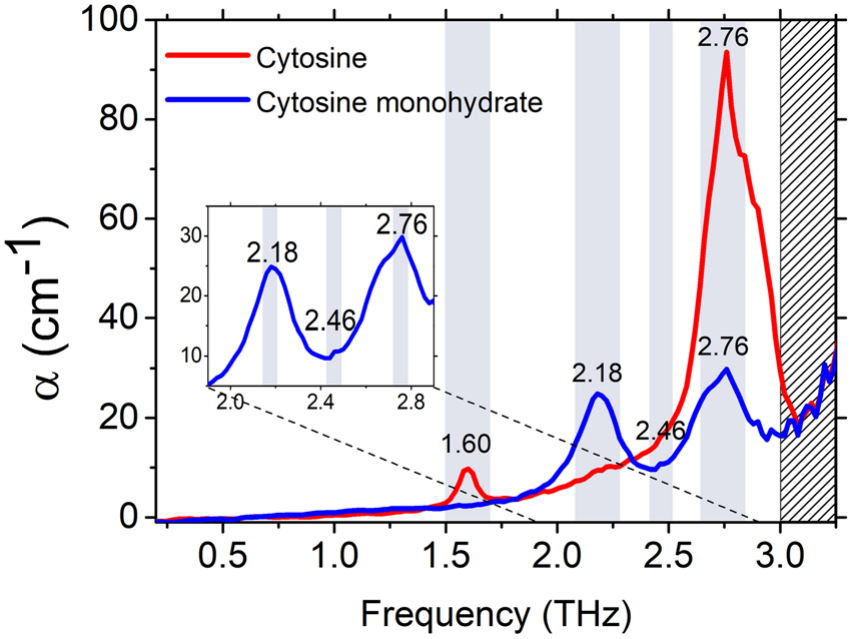
THz spectra of C and C-MH. C has an absorption peak at 1.60 THz where the C-MH peak was absent. C-MH has new peaks at 2.18 and 2.46 THz. Both samples have 2.76 THz peaks of different intensities. The small figure is the magnified graph of C-MH in the region between 1.90 to 2.90 THz.

The experimentally acquired absorption coefficients of C and C-MH were compared in the 0.2–3.0 THz range, as shown in Fig. 1. The overall exponential background baseline was the scattering effect influenced by the particle size of polyethylene powder, the material used to make stable pellets with low absorptivity in the THz region^31^. The C displayed spectral peaks at 1.60 and 2.76 THz. The peak of 1.60 THz showed an intensity of 9.75 cm^−1^, while the peak of 2.76 THz had a large intensity of 93.6 cm^−1^. The experimentally acquired spectral THz peak positions and absorption levels of C and C-MH, at room temperature, are summarized in Table 1. Our absorption peak at 1.60 THz was the same value as in previous studies, however, that at 2.76 THz differed slightly. The variation of the 2.76 THz peak may have been affected by the particle size, drying time, and disk composition, which are known to influence peak positions^14,32^.

**Table 1:** The THz absorption peaks of C powder at room temperature in various studies.

Sample	C				C-MH
	This work	Ref. ^55^	Ref. ^56^	Ref. ^57^	This work
**THz-TDS experiments**					
Main peaks (absorption level)	1.60 THz (9.75 cm^−1^)	1.6 THz	1.6 THz	1.59 THz	2.18 THz (24.86 cm^−1^)
	2.76 THz (93.56 cm^−1^)	2.7 THz	2.85 THz	2.73 THz	2.46 THz (10.74 cm^−1^)
		3.3 THz	3.39 THz		2.76 THz (29.83 cm^−1^)

The THz absorption spectrum of C-MH was investigated first in this study. The C-MH spectrum had a different absorption spectrum to C because THz regions have unique absorption peaks according to molecular conformations^33,34^. In the C-MH spectrum, a noticeable peak appeared at 2.18 THz, with an intensity of 24.9 cm^−1^. Additionally, a very small new peak at 2.46 THz was observed, which was almost obscured by two peaks at 2.18 and 2.76 THz. The absorption peak at 2.76 THz had an intensity of 29.8 cm^−1^, which was a lower amplitude compared to the intensity of 93.6 cm^−1^ at the same frequency of the C spectrum. The peak amplitudes of 2.18 and 2.76 THz had a similar height in the C-MH spectrum. The difference in the absorption spectra of C and C-MH was due to the incorporation of water molecules, which contribute to the crystal structures and intermolecular interactions via hydrogen bonds. The THz spectra obtained by THz-TDS can effectively identify C and C-MH with distinct peaks, which suggests that this technique can potentially be applied to the pharmaceutical industry to distinguish between unexpectedly hydrated ingredients^33–39^. To further investigate the relation between the crystal structure and THz spectrum, we performed PXRD and geometry optimization using DFT simulations.

### Crystal structure and geometry optimization

**Figure 2:**
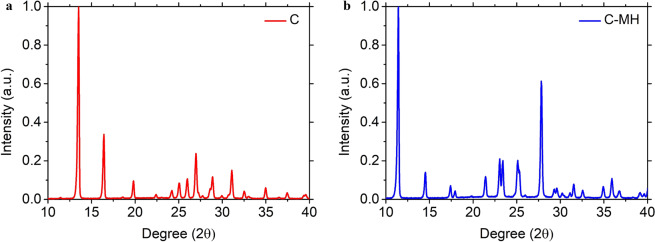
The PXRD patterns of C and C-MH. The graph (**a**) and (**b)** represents the XRD patterns of C and C-MH in the range of degrees of 10 to 40 in 2θ. It shows that they have different crystalline structures.

**Figure 3:**
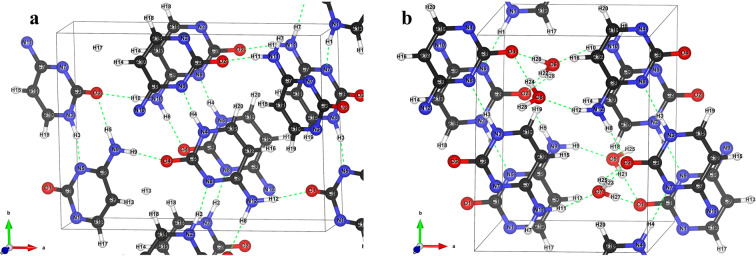
The crystal structure of (**a**) C and (**b**) C-MH. The black, white, blue, and red atoms represent carbon, hydrogen, nitrogen, and oxygen atoms, respectively. The black line refers to the crystal axes. The green dotted lines represent hydrogen bonds.

PXRD is a well-known experimental technique, used to extract information on the crystal structure. The diffraction patterns of the neat grounded samples of C and C-MH are shown in Fig. 2a,b. The PXRD pattern showed that the two crystal structures had distinct diffraction characteristics. Lattice constants extracted from the PXRD pattern were compared to the lattice constants from other studies, as displayed in Table 2. C has orthorhombic P212121 space group symmetry with 4 molecules in each unit cell. The lattice constants are a = 13.044,b = 9.507, and c = 3.820. C-MH has monoclinic P21/c space group symmetry with four C molecules and four H_2_O molecules. The lattice constants are a = 7.795 Å, b = 9.838 Å, c = 7.684 Å, and ${\boldsymbol{\beta }}$ = 99.495°. The lattice parameters between ref. ^40^ and our data are in agreement by more than 99.8% for C and 99.7% for C-MH. The similarity of the lattice constant to previous research indicates that the sample we recrystallized had the same crystalline structure as that used in the published paper. The atomic positions acquired with Rietveld refinement were fully relaxed with the density functional theory using plane-wave self-consistent field programs in Quantum ESPRESSO (QE)^41,42^. This was done to investigate the hydrogen bond environment in molecular geometry, which was related to the vibrational modes in the THz region. The optimized crystal structure lattice parameters were very close to experimental results and are shown in Table 2. The fully relaxed crystal structure is shown in Fig. 3a,b. The compositions and lengths of the hydrogen bonds are described in Table 3.

**Table 2:** Lattice constants of C and C-MH.

Lattice constant	C			C-MH		
	Exp.^a^	Exp.*	Cal.*	Exp.^a^	Exp.*	Cal.*
a (Å)	13.044	13.044	12.888	7.783	7.795	7.641
b (Å)	9.496	9.507	9.430	9.825	9.838	9.737
c (Å)	3.814	3.820	3.613	7.668	7.684	7.277
α (°)	90	90	90	90	90	90
β (°)	90	90	90	99.57	99.495	101.044
γ (°)	90	90	90	90	90	90
V (Å)^3^	472.424	473.68	439.131	578.196	581.18	531.386

^a^The lattice constant is from ref. ^40^. ^*^This work.

**Table 3:** The distances of hydrogen bonds in C and C-MH.

C		C-MH	
Hydrogen bond	Distance (Å)	Hydrogen bond	Distance (Å)
N1-H1 … N7	1.703	N1-H1 … N6	1.811
N9-H5 … O3	1.893	N9-H5 … O2	1.860
N9-H9 … O4	1.873	N9-H9 … O5	1.858
		O1 … H21-O5	1.781
		O1 … H27-O7	1.735

The incorporation of water molecules primarily affects the hydrogen bond network in C, including the crystal structure, atomic positions, and intermolecular vibrations, which lead to a change in the characteristic THz spectra of the C molecules. The hydrogen bond strength is considered to correlate to the length of the hydrogen bond^43^. The hydrogen bond between the amine group and the nitrogen in the pyrimidine of C-MH (N1-H1⋯N6) was weaker than that of C (N1-H1⋯N7) due to the increase in the hydrogen bond length from 1.703 Å to 1.811 Å. However, the two types of hydrogen bonds by two hydrogen atoms of the amine group with the ketone groups decreased from 1.893 and 1.873 Å in C to become one type of hydrogen bond with a length of 1.860 Å in C-MH. Additionally, C-MH had three types of strong hydrogen bonds that were newly generated between C and the water molecules. The amine group formed a hydrogen bond of length 1.858 Å with water-bound oxygen. The lengths of the hydrogen bonds between the C ketone groups and the hydrogen atoms of water molecules were 1.781 Å for O1⋯H21-O5 and 1.735 Å for O1⋯H27-O7. The C-MH hydrogen bonds were stronger and more abundant than those of C, which leads to different intermolecular interactions of C molecules and a more stable crystal structure with new cell parameters, i.e., the co-crystallization process^12,44^. Also, a change in the molecule’s environment and conformation leads to a characteristic change in the THz spectra. The represented THz vibrational modes of C and C-MH can be identified by simulating normal modes using these optimized crystal structures.

### Simulated THz spectra using density functional theory

**Figure 4:**
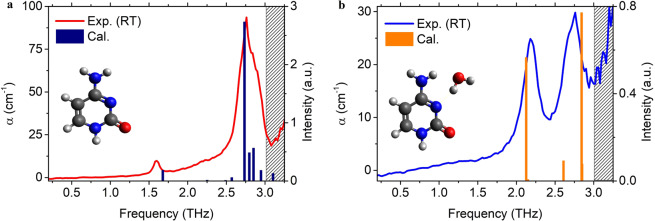
Experimental and theoretical THz absorption spectra of (**a**) C and (**b**) C-MH. The simulated normal modes were scaled by 0.88 of C and 0.85 of C-MH.

The normal modes of the samples were calculated, using the density-functional perturbation theory in the Phonon package of QE, to investigate the vibrational modes of the C and C-MH peaks in THz spectra^41,42^. The fully relaxed geometry was used to calculate the phonon normal modes with a dynamical matrix, dielectric constant, and Born effective charges, using interatomic force constants obtained by finite perturbation within the DFT framework. The infrared (IR) intensity was calculated with the effective charges and phonon displacement patterns within QE. The details of the calculation are described in ref. ^45^. The calculated spectra were compared to the experimental results in Fig. 4, within the range of 0.2 to 3.0 THz. The predicted vibrational frequencies of the simulated DFT results were over-estimated compared to the experimental results. This is because the experiments were performed at 294 K while the simulations are performed at 0 K, which leads to the shift to higher-frequencies upon cooling. The linear scaling factors were adjusted to all vibrational modes of C and C-MH, with scaling factors of 0.88 and 0.85, respectively^46,47^. The reason for the more scaled factor of C-MH might be the vibrational anharmonicity which plays a significant role in H_2_O-containing molecular crystal^48^.

In the absorption spectrum of C (Fig. 4a) the small experimental peak at 1.60 THz represented a peak calculated at 1.68 THz. The large peak at 2.76 THz represented multiple normal modes of 2.73, 2.80, and 2.85 THz. The simulated modes at 2.73 THz significantly contributed to the experimental result at 2.76 THz. The calculated relative intensities of the normal modes were in good agreement with the experimental absorption coefficients at 1.60 and 2.76 THz. In the case of C-MH (Fig. 4b), the absence of the 1.6 THz peak, shown for C, was well reproduced by the calculation of the normal modes of C-MH. Additionally, the discovered peaks at 2.18 and 2.46 THz, from the experiments, were represented by 2.13 and 2.61 THz, respectively, and the peak at 2.76 THz was reproduced with multiple modes at 2.84, and 2.85 THz. Compared to the case of C, the peak at 2.76 THz in the THz spectrum of C-MH appeared with fewer vibrational modes and lower intensity, which was similar to the experimental results. In summary, the theoretical calculation using DFT appropriately replicated the low-frequency experimental results for both samples. However, to better understand the vibrational modes of each peak, the corresponding mode analysis is still required.

## Discussion

**Figure 5:**
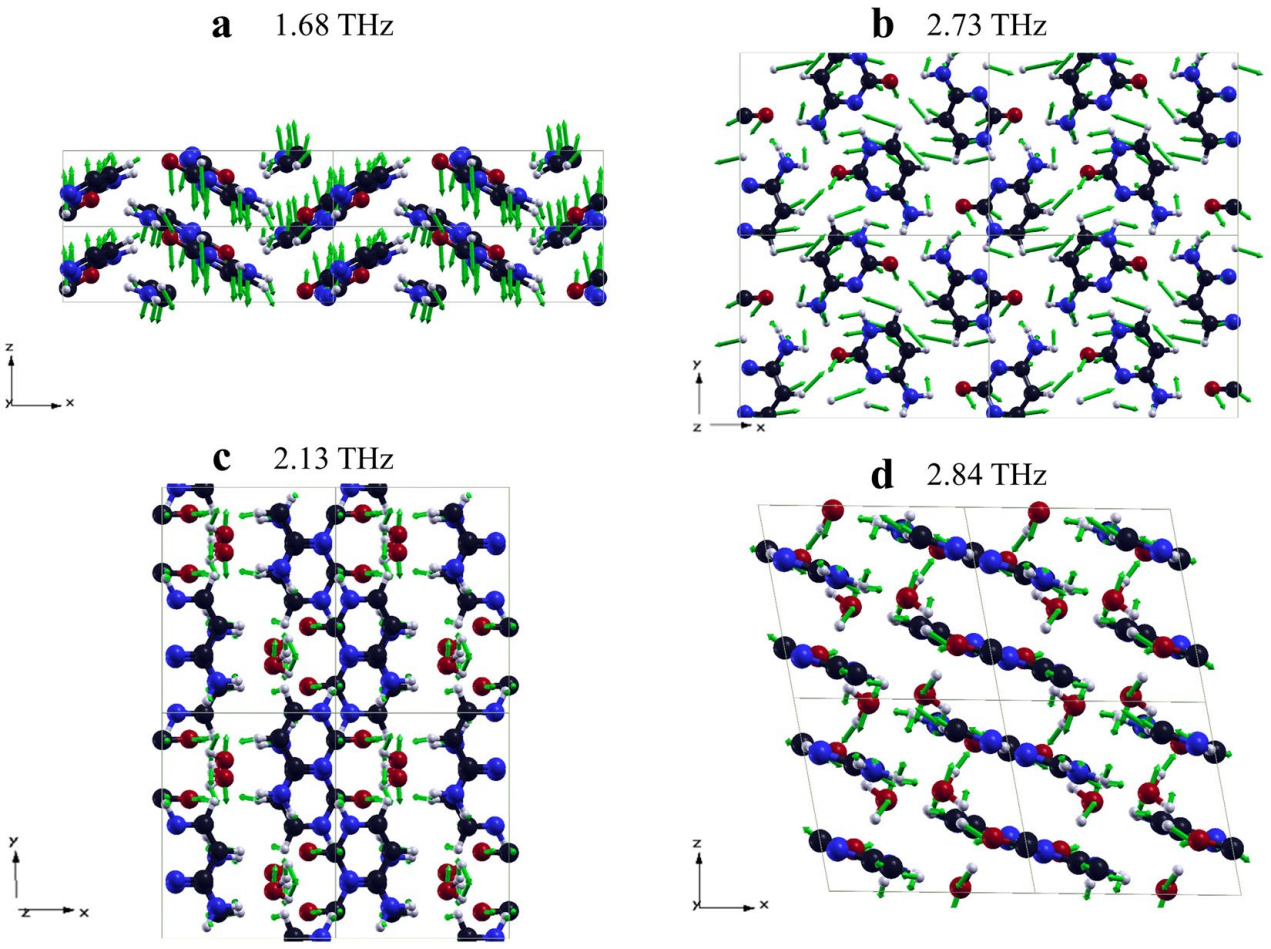
Visualization of major vibrational modes of C and C-MH (**a**) 1.68 THz and (**b**) 2.73 THz of C, and (**c**) 2.13 THz and (**d**) 2.84 THz of C-MH. The black, white, blue, and red atoms represent carbon, hydrogen, nitrogen, and oxygen atoms, respectively. The grey line refers to the crystal axes. The green arrows show the displacement vector of each atom.

We visualized the major molecular normal modes in Fig. 5 using XCrySDen, a crystalline and molecular structure visualization program^49^. We also performed the mode character analysis for a single molecular basis to quantitatively identify intermolecular and intramolecular vibrations. We separated the main vibrational modes into intermolecular and intramolecular vibrations following the method organized in ref. ^50^ using Cartesian coordinate atomic positions and displacement vectors. The center of mass displacements along the crystallographic Cartesian coordinate three axes were used to express three intermolecular translations along the three axes at each mode. We then transformed the atomic positions and displacement vectors into the principal axis to acquire intermolecular libration, which is the reciprocating rotational motion of the molecule as a rigid body. The libration angles were computed by dividing the whole sum of the cross product between each atomic positions and each displacement vectors in the principal axes by each principal moment about each axis. The displacement vectors of principal libration about the three principal axes were collected by subtracting the transformed positions vectors from rotated position vecors about libratoin angle. Thereafter the intermolecular librations along the ordinary three axes were acquired by retransforming the principal molecular axes into the crystallographic Cartesian coordinate system. The intramolecular vibrations of each molecule at each mode were collected by subtracting the three intermolecular translations and three intermolecular librations at total displacements. The relative percentage contributions of all intermolecular and intramolecular vibrations of each molecules were calulcated by root-mean-square mass-weighted displacements by the following equation.:

$${{\rm{P}}}_{{\rm{Trans}},{\rm{X}}}=\frac{\sqrt{\frac{1}{{\rm{O}}}{\sum }_{i}^{{\rm{O}}}{m}_{i}{\delta }_{i,Trans,X}^{2}\,}}{\sqrt{\frac{1}{{\rm{O}}}{\sum }_{i}^{{\rm{O}}}{m}_{i}{\delta }_{i,Trans,V}^{2}}+\sqrt{\frac{1}{{\rm{O}}}{\sum }_{i}^{{\rm{O}}}{m}_{i}{\delta }_{i,Lib,V}^{2}}+\sqrt{\frac{1}{{\rm{O}}}{\sum }_{i}^{{\rm{O}}}{m}_{i}{\delta }_{i,Intra}^{2}\,}}\times 100 \% ,\,V=X,Y,Z$$

where ${m}_{i}$ refers to relative atomic mass, ${\rm{O}}$ refers to the number of atoms in a molecule, V refers to three-axis and ${{\rm{\delta }}}_{{\rm{i}},{\rm{Trans}}},\,{{\rm{\delta }}}_{{\rm{i}},{\rm{Lib}}},\,{\rm{and}}\,{{\rm{\delta }}}_{{\rm{i}},{\rm{Intra}}}$ refer to translational, librational, and intramolecular displacements. Finally, we averaged each molecule’s relative mode contributions in the unit cell to obviously distinguish the relative vibrational contribution from other modes.

**Figure 6:**
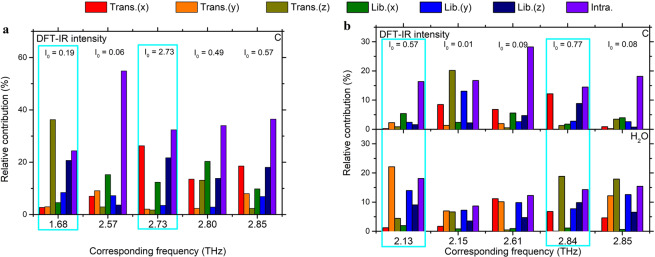
Mode characteristics of five major vibrational modes of (**a**) C and (**b**) C-MH in the range of 0.2 to 3.0 THz. The boxes of cyan color represent the most contributing normal modes to the experimental THz resonance peaks.

Fig. 6a,b show the five main vibrational modes that appeared in the THz region of C and C-MH. The overall mode characteristics of C and C-MH in the low-frequency region were identified by mixed intermolecular and intramolecular vibrations in most cases.

In Fig. 6a, the intramolecular vibrations increase from 24.36% at 1.68 THz to 36.46% at 2.85 THz except for one mode at 2.57 THz with a large contribution of 54.88%. Among the five major vibrational modes, There were two modes of 1.68 and 2.73 THz which contribute the most to the experimental resonance peaks at 1.60 and 2.76 THz. The experimental vibrational mode of C at 1.60 THz was denoted to have 36.25% of the translations along the z-axis with 24.36% of the intramolecular vibrations and 20.68% of the librations about the z-axis at 1.68 THz. Figure 5a shows the view of C’s translations along the z-axis. Figure 5b represented the vibrational modes 2.73 THz corresponding to the absorption peak at 2.76 THz of C. The mode characteristics corresponded to 26.26% of the translation along the x-axis and 21.69% of the librations about the z-axis which is quite similar shown in Fig. 5b. Figure 6b presents the mode contributions of C-MH showing that the intermolecular vibrations of H_2_O affected its THz normal modes more than about 50% in major vibrational modes except 2.15 THz which is small IR intensity compared to the other modes. There were two simulated normal modes of 2.13 and 2.84 THz to most contributing to experimental THz peaks of 2.18 and 2.76 THz. The simulated 2.13 THz normal mode mostly consists of 22.13 % translation along the y-axis of water molecules. It was verified in Fig. 5c, which shows the y-axis translation of water molecules and also other contributions. Figure 5d displays the translational movement along the declined z-axis of the water molecules and the motions of cytosine atoms within the tilted xy-plane at 2.84 THz. In Fig. 6b, this mode denoted 18.84% translation along the z-axis of the water molecules and 12.15% translation along the x-axis of the C molecules with approximately 14% of intramolecular vibrations of both molecules, but also librations about the z-axis of both water and C molecules at 9.84 and 8.85% respectively.

In comparison with the mode characteristics of C molecules in crystal C and C-MH, the modes of C molecules had distinct characteristics of intermolecular and intramolecular vibrations in most cases caused by the different molecular conformations with water molecules. However, there were the simulated modes that have considerably common-mode features at the mutual experimental peak of 2.76 THz at room temperature, even though C and C-MH have different intermolecular interactions by hydrogen bonds. The DFT simulation results of C and C-MH represented the 2.76 THz experimental peaks as the 2.73 and 2.84 THz, respectively. The relative mode contributions appeared in common that the C molecules have the translation along the x-axis, libration about the z-axis, and intramolecular vibration except for the libration about the x-axis. Based on this aspect, the hydrogen bonds between C and H_2_O not only lead to the different crystal structures and low-frequency spectra but also the partial or overall changes of mode characteristics of C molecules.

Our DFT simulation showed that the low-frequency normal modes calculated with QE were in good agreement with the THz absorption spectra of C and C-MH. To identify the calculated peaks of small intensities that were not clearly observed in the experimental spectra, further study is required, using a low-temperature THz-TDS system. However, the simulation results improve our understanding of the origins of normal modes in the low-frequency region. The THz-TDS which uses DFT calculation and mode analysis is a robust technique to investigate how water molecules contribute to each low-frequency molecular vibrational mode, which is consisted of the collective vibrations mixed with intermolecular and intramolecular vibrations affected by hydrogen bonds.

We investigated how the addition of water molecules in C affects the THz spectra by obtaining the low-frequency absorption spectra of C and C-MH, using THz-TDS. The crystal geometries were analyzed with the PXRD technique to identify the crystal structure that influenced the different THz spectra and the hydrogen bonding environments were investigated using full relaxed atomic positions acquired by DFT calculations. The normal modes calculated using DFT agreed well with the experimental absorption peaks in the THz range, illustrating the origin of different THz spectra. The low-frequency vibrational modes of C and C-MH were represented by mixed intermolecular and intramolecular vibrations. The mode characteristics of experimentally observed THz peaks of C originated mostly from the intermolecular translation of C molecules and those of C-MH originated mostly from the intermolecular translation of water molecules contributed to the THz resonance peaks. And we can discriminate the transformation of most intermolecular vibrations of C molecules in both crystals which helped to understand how water molecules contribute to the low-frequency vibrational modes by intermolecular vibrations with hydrogen bonding. This result suggests that THz-TDS combined with DFT simulations and mode analysis can be utilized to not only identify the molecules but also understand the origin of intermolecular interactions in the biological, biomedical, and pharmaceutical applications.

## Methods

### Materials and sample preparation

The Cytosine (C) was purchased from Sigma-Aldrich with the purity > 99 %. The cytosine monohydrate (C-MH) was produced by exposing a 100-mg sample of C in a chamber with 0.5 ml distilled water at room temperature for a few days. These samples were used without further purification. To obtain a clear THz absorption spectrum, the samples were mixed with polyethylene powder at a 1:10 mass ratio, and grounded with a mortar and pestle for 5 min. The samples were compressed at 2000 psi for 10 min to produce pellets that had a thickness of 500–600 µm and a diameter of 1.2 mm.

### Terahertz spectroscopy

We used a Ti:sapphire laser (Synergy; Spectra-Physics, USA), with a wavelength of 800 nm, a pulse duration of 10 fs, and an 80 MHz repetition rate. The laser was pumped by a Verdi 10 W diode laser. The launched laser beam was separated by a beam splitter to form the generation and probe beams. The generation beam was incident on p-InAs at an angle of 78° to generate the THz pulse, as a result of photo-Dember effects. The generated THz pulse was focused on the sample pellet using a pair of THz lenses (Tsurupica; Microtech Instrument, Inc., OR, USA). The THz pulse transmitted through the sample was reflected by a parabolic mirror and focused by a silicon lens onto the 5 µm gap photo-conductive antenna (PCA, TERA8-1; MenloSystems, Germany). The attenuated probe beam generated the photocarriers, and the THz pulse induced a current in the PCA gap. The current was amplified by lock-in amplifiers (SR 850, Stanford Research System, USA) and recorded in the time-domain by successively controlling the moving stage incrementally. The pathway of the THz pulse was contained in a chamber filled with nitrogen gas, to reduce the attenuation of the THz signal by water vapor. The time-domain waveforms of the reference, C, and C-MH were recorded and the absorption coefficients were extracted using a Duvillaret numerical algorithm^51^.

### Powder X-ray diffraction analysis

The PXRD pattern was measured with a Cu Kα line (1.54 Å), at a voltage of 40 kV and a current of 200 mA, using a Dmax 2500 (Rigaku, Japen). The data was collected with a scan range from 10° to 90° (2θ), and the scan range from 10° to 40° was used to show a clear difference in the data. The scan width was 0.02° and the scan speed was 2°/min. The lattice parameters were determined by Rietveld refinement using the initial model of ref. ^40^. Rietveld refinement was performed within the TOPAS (Bruker AXS GmbH, Germany) program. The background was fitted by the Chebychev polynomial functions. Modified pseudo-Voigt functions were used as shape functions. All atomic positions were refined except for those of the hydrogen atoms.

### Density functional theory calculations

All theoretical calculations for geometry optimization were performed with plane-wave self-consistent field code of quantum ESPRESSO (QE), which is a calculation software using a plane-wave basis set and pseudopotentials within density functional theory (DFT)^41,42^. Projector-augmented wave pseudopotential was utilized in the simulation with the Perdew-Burke-Ernzerhof exchange-correlation functional^52^. The van der Waals correction of ‘Grimme-d2’ in the QE package was adjusted in all geometry optimizations^53^. The total energy and force convergence threshold for ionic minimizations were 1.0 × 10^−5^ Ry/atom and 5.0 × 10^−6^ Ry/atom, respectively. The kinetic energy cutoff for wavefunctions and for charge density and potential were chosen as 60 Ry and 600 Ry, respectively. The convergence threshold for iterative calculations of the self-consistent field was 1.0 × 10^−10^ Ry. Monkhorst-Pack k-point samplings of a 4 × 4 × 4 grid were used to sample the Brillouin zone for both the C and C-MH. The geometry optimization was conducted using the structure obtained by Rietveld refinement from our XRD results. Both, atomic positions and cell parameters were fully relaxed. The geometry of both samples was identified and visualized with the Visualization of the Electron/nuclear and Structures software, a 3D visualization program for structural models^54^

Reprints and permissions information is available at www.nature.com/reprints. The authors declare no competing financial interests. Readers are welcome to comment on the online version of the paper. Correspondence and requests for materials should be addressed to J.–H. Son (joohiuk@uos.ac.kr).
